# Are interpersonal communication skills adequately taught at postgraduate specialist level in South Africa? The neurology experience

**DOI:** 10.4102/safp.v63i1.5275

**Published:** 2021-06-15

**Authors:** Anand Moodley, Anton van Aswegen, Liesl Smit

**Affiliations:** 1 Department of Neurology, Faculty of Health Sciences, University of the Free State, Bloemfontein, South Africa; 2 Department of Neurology, Universitas Hospital, Bloemfontein, South Africa; 3 Department of Neurosurgery, Faculty of Health Sciences, University of the Free State, Bloemfontein, South Africa

**Keywords:** interpersonal communication, AfriMEDS, CanMEDS, breaking bad news, disclosing medical errors, objectively structured clinical exam, objectively structured practical exam

## Abstract

**Background:**

Interpersonal communication skills by clinicians with patients, carers, fellow health professionals and legal professionals carry many unique challenges in practice. Whilst undergraduate training in communication helps with generic information receiving and information giving, uncomfortable and demanding speciality-specific issues in the various medical specialities are not covered during under- and postgraduate training.

**Methods:**

The aim of this study was to determine the self-perceived competence of neurology registrars and neurologists in interpersonal communication and the need for such assessment in college exit exams. We undertook a quantitative, descriptive, cross-sectional survey by using self-administered printed questionnaires and the EvaSYS online system. Neurology registrars in training from the seven training centres in South Africa and neurologists based at the training centres and in the private sector were recruited.

**Results:**

We received a 62.9% response rate. One hundred and twenty-nine participants were recruited comprising 42 neurology registrars and 87 neurologists. Registrars were more commonly female, more likely to be multilingual and less likely to use translators. Undergraduate training in communication was considered insufficient, 42.9% and 39.1% for registrars and specialists respectively, and was also considered not relevant to address speciality-specific issues encountered in practice. Most training received has been by observation of others and on-the-job training. Both groups felt strongly that postgraduate training in interpersonal communication was important (registrars 95.2%, specialists 91.9%), especially when dealing with issues of death and dying, disclosing medical errors and dealing with the legal profession.

**Conclusion:**

Postgraduate training of interpersonal communication as required of neurology registrars and neurologists was considered insufficient. Most training has been by observation of others or experiential by trial and error. Assessment of interpersonal communication at board exit exams will drive postgraduate training and importantly will embrace the AfriMEDS framework developed to produce the holistic doctor in South Africa.

## Introduction

Counselling of patients faced with bad news requires a set of skills beyond the examination skills most clinicians are trained in and are expected to be experts at performing. Breaking bad news, disclosing medical errors, dealing with distressed patients and family and communicating with fellow health professionals and legal professionals require adept communication skills.^1,2^ Depending on the clinician’s abilities, he or she either shies away from these vexing encounters or performs poorly with potential and unfortunate legal consequences.^3^ It is not uncommon for patients to feel abandoned by their doctor when communication is poor.^4^ Not all doctors are inept in interpersonal communication, but the stark reality in South Africa is that communication in medicine is mostly self-taught, dependent on learning on-the-job or learnt from mishaps from the past. Improved communication between the doctor and patient results in an improved therapeutic relationship and better management outcomes.^1,3,4^ Undergraduate communication training and assessment are formalised in South African medical schools, but postgraduate training in communication is not. Specialist training units abroad offer interpersonal communication training as an essential component in training. In Canada, the Canadian Medical Education Directives for Specialists (CanMEDS) framework for resident training is a requirement both in the training and assessment of doctors.^5^ The CanMEDS framework for physicians training considers the healthcare worker to be a medical expert with all the clinical skills and medical knowledge required to practise. In addition, the healthcare worker requires training in specific skills to be a communicator, collaborator, manager, health advocate, scholar and professional to allow for the holistic practice of medicine. In the United Kingdom (UK), the objectively structured clinical exams (OSCEs) include the assessment of interpersonal communication stations during under- and postgraduate exams.^6^

Neurology as a speciality presents with many challenging, uncomfortable and demanding communication issues in clinical practice.^7^ The nature of the profession is such that often degenerative, disabling and non-remitting disorders are diagnosed in patients across all generations. The South African neurology curriculum required for training in neurology is outlined in the College of Neurology website.^8^ It is intended for use by the various training units as well as current and future neurology registrars. The emphasis of training is on the gaining of clinical knowledge and the acquisition of clinical skills in the fields of neurology and neurophysiology. Upon the completion of 4 years of training in neurology at an accredited training unit in South Africa, a successful outcome in the parts one and two neurology board exams and a successful completion of an MMed dissertation, the candidate becomes eligible for a licence to practise neurology independently in South Africa. The focus is mainly on theoretical neurological knowledge and practical neurology skill acquisition for the board exam. Research skills for the MMed dissertation have been a recent addition. However, interpersonal communication is neither offered in training nor assessed in the board exit exams. Candidates are expected to know this from undergraduate training.

Personal experience shows that the doctor–patient, as well as interpersonal communication with fellow health professionals, is below par. But this is subjective and not representative of the whole specialist fraternity. It is likely that the 4 years of training is sufficient for all aspects of training and that additional communication training might be an overindulgence. Regardless of this, a consensus view is necessary to gauge the competency of specialists and registrars in interpersonal communication and to inform the relevant colleges about the need for additional training and assessment if needed.

As a neurologist, I understand the communication demands of this speciality, hence the decision to study the postgraduate training in communication received by neurologists. Furthermore, neurology is a small discipline with the perception of greater trainer–trainee contact and perhaps more interpersonal communication training. We therefore undertook a paper-based and online survey of registrars and specialists in the public and private sectors to gauge their opinions regarding interpersonal communication training in South Africa.

The aim of the study was to assess the self-perceived competence of registrars and graduates in interpersonal communication and to elicit their perceptions on the importance of interpersonal communication as a core competency requirement in the training and assessment of specialist neurologists. The secondary aim was to estimate the willingness of neurology doctors to participate in communication training at various levels of training and post-training.

## Method

A quantitative, descriptive, cross-sectional survey was used. A minimal qualitative aspect was included when additional suggestions from those listed were requested. A self-administered paper-based questionnaire and online survey by using the EvaSys system provided by the University of the Free State (UFS) were distributed to all neurology registrars and specialists in the public, academic and private sectors.^9^ There are currently about 45 registrars and 160 qualified neurologists in the country.^10^ The survey was conducted over a 4-month period from 01 February 2020 to 31 May 2020. Registrars were recruited from the seven neurology training units in the country located at the Universities of the Free State, KwaZulu-Natal, Cape Town, Witwatersrand, Pretoria, Stellenbosch and Sefako Makgatho. Specialist neurologists were recruited from these training units and from various cities and towns where their private practices are located. The online link for the EvaSys system was submitted via email and via the WhatsApp messenger service. Email addresses were obtained from the Neurological Association of South Africa (NASA) database with the expressed approval of the NASA executive. The EvaSys link was sent via a message on the NASA WhatsApp group administered by the president of NASA. Registrars were recruited at the annual registrar teaching weekend and via email addresses also obtained from NASA. The required number of registrars and specialists to be sufficiently representative was 40 and 60, respectively.

### Data analysis

Support for data analysis was provided by the UFS biostatistics department. Results were summarised as frequencies and percentages (categorical variables) and means, standard deviations or percentiles (numerical variables); 95% confidence intervals were calculated for main outcomes. The Fisher’s exact test and Chi squared tests were performed for comparative data.

### Ethical considerations

Ethics approval for the study was obtained from the UFS human sciences research ethics committee (ethics approval clearance number: UFS-HSD2020/0028/2605).

## Results

One hundred and twenty-nine participants responded to the survey, amounting to a 62.9% response rate. This high response rate was achieved by sending the EvaSys online survey link by email and by the active NASA WhatsApp messaging group. There were 42 registrars and 87 neurology specialists who participated. Table 1 shows the demographic data of this cohort. As expected, the members of the specialist group were older and had more years of experience. The trend informally adopted by most universities of recruiting more female registrars especially in the medical disciplines was reflected in the higher percentage of female registrars (62%) who participated. The higher percentage of qualified male specialists who participated (60%) (*p* = 0.023) reflects the previous gender distribution; however, there are no current or previous statistics available to confirm this distribution. See Table 1.

**TABLE 1:** Demographic data, current location, training units and language proficiency of participants.

Variable	Registrars				Specialists				Total		Fisher Exact *p*-value
	*n*	%	Median	IQR	*n*	%	Median	IQR	*N*	%	
**Age**	-	-	33	-	-	-	47	-	-	-	-
Range	28–51	-	-	-	32–79	-	-	-	-	-	-
**Gender (male)**	16	38	-	-	52	-	-	-	-	-	0.023
**No. of years of experience**	-	-	2	1;3	-	-	19	11;29	-	-	< 0.05
**Current location**											
KwaZulu-Natal	10	24	-	-	27	32	-	-	37	29	-
Free State Province	3	7	-	-	4	5	-	-	7	6	-
Western Cape	10	24	-	-	22	26	-	-	32	25	-
Eastern Cape	0	-	-	-	1	1	-	-	1	1	-
Gauteng	18	44	-	-	31	36	-	-	49	39	-
Total (*N*)	41	-	-	-	85		-	-	126		-
**University training unit**											
Free State	3	7	-	-	5	6	-	-	8	6	-
Witwatersrand	11	26	-	-	22	27	-	-	33	27	-
Pretoria	3	7	-	-	12	15	-	-	15	12	-
Sefako Makgatho	5	12	-	-	1	1	-	-	6	5	-
Cape Town	5	12	-	-	7	9	-	-	12	10	-
Stellenbosch	5	12	-	-	11	13	-	-	16	13	-
KwaZulu-Natal	9	21	-	-	24	29	-	-	33	27	-
Other	1	2	-	-	0	-	-	-	1	1	-
Total (*N*)	42	-	-	-	82		-	-	124	-	-
**Language proficiency**											
English	35	83	-	-	86	99	-	-	121	94	0.0016
Zulu	6	14	-	-	3	3	-	-	9	7	0.0576
Afrikaans	12	29	-	-	52	60	-	-	64	50	0.0013
Xhosa	3	7	-	-	0	-	-	-	3	2	-
Sesotho	5	12	-	-	1	1	-	-	6	5	-
Venda	1	2	-	-	0	-	-	-	1	1	-
Tswana	4	10	-	-	0	-	-	-	4	3	-
Tsonga	1	2	-	-	0	-	-	-	1	1	-
Siswati	0	-	-	-	0	-	-	-	0	-	-
Ndebele	1	2	-	-	0	-	-	-	1	1	-
Northern Sotho	5	12	-	-	0	-	-	-	5	4	-
Total (*N*)	42	-	-	-	87	-	-	-	129	-	-

Note: Some registrars and neurologists were proficient in more than one language.

Registrars: *n* = 42, 33%; Specialists: *n* = 87, 67%.

The larger cities provided more registrar and specialist participation, where more doctors are clustered.

Given that English is the common medium of instruction at all universities in South Africa, 94% of participants reported proficiency in English. English and Afrikaans were most spoken by registrars and specialists, but significantly more by the specialists (Fisher’s exact score *p* = 0.0016 and 0.0013, respectively). Zulu was spoken more by registrars, showing a trend but not statistical significance on the Fisher’s exact score (*p* = 0.0576).

Registrars showed a more diverse language proficiency in the other ethnic official languages and were less likely to use interpreters when communicating with patients (56% of registrars vs. 92% of specialists, *p* < 0.0001). Both groups faired equally well with their perceived competency in communicating with other healthcare professionals and patients, despite specialists using translators more often (Appendix 1, Figure 1-A1). Neither group saw their language skills as a barrier to their communication.

Both groups reported that they had been inadequately trained for interpersonal communication with patients and other healthcare professionals. More registrars than specialists received communication training during the undergraduate years (71.4% vs. 26.4% *p* < 0.0001), but they were in agreement that neither group had adequate communication training during postgraduate education (registrars 83.3%, specialists 83.9%, *p* = 1.000).

For those who experienced interpersonal communication training during their undergraduate years, most training was conducted in the fourth year of study for registrars in the form of lectures and role playing but was equally distributed during the first five years for specialists (Figure 1). The focus of training has been on interpersonal communication for history taking and information giving but not really with regard to problematic management issues. Some communication training with families were offered to registrars. Communication with other healthcare professionals, communication in writing and especially communication with the legal professionals were most unsatisfactory (Figure 2).

**FIGURE 1:**
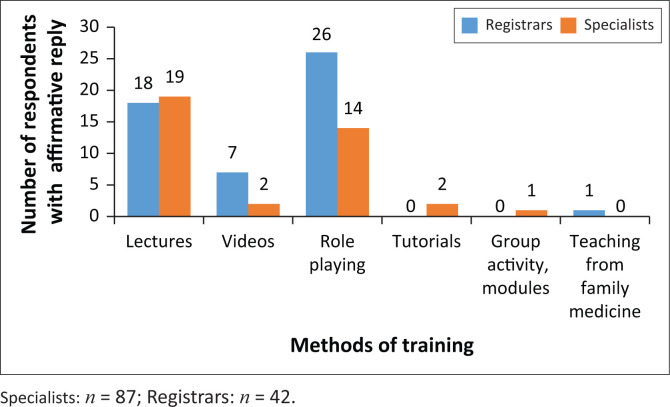
Type of training received at undergraduate level.

**FIGURE 2:**
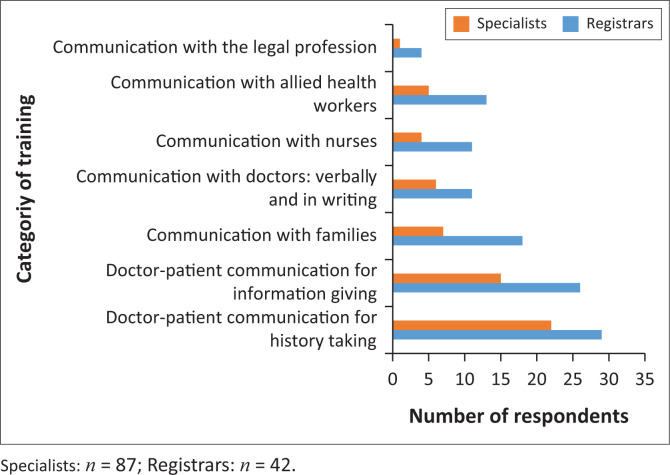
Category of training received at undergraduate level.

Doctor–patient communication was tested formally for most registrars at undergraduate level but not for specialists (registrars 69.1% vs. specialists 23.3% *p* < 0.0001). For those who did have formal testing, the format was mostly role playing. A formal written exam was also a means of testing reported by both registrars and specialists. Three registrars reported testing of communication skills by OSCEs and two more registrars had videos shown to them followed by a written test.

For those who did receive undergraduate communication training, there was a tendency by specialists to feel that the training they received did not prepare them adequately for practice (Table 2). Registrars and specialists did not feel competent in neurological communication issues and felt strongly that training was important (registrars 95.2%, specialists 91.9%, *p* = 0.08). Both groups also felt strongly that interpersonal communication does not belong to the realm of psychiatry alone.

**TABLE 2:** Opinions relating to interpersonal communication training.

Variable	Registrar						Specialist						Fisher’s exact score *p*

	Yes		No		Unsure		Yes		No		Unsure		
	*n*	%	*n*	%	*n*	%	*n*	%	*n*	%	*n*	%	
Did undergraduate communication training prepare you for practice?	16	53.3	12	40.0	2	6.7	6	26.1	16	69.6	1	4.4	0.09
Do you feel competent in neurology communication issues?	22	52.4	13	31.0	7	16.7	47	54.7	28	32.6	11	12.8	0.83
Is formal training in doctor–patient communication important?	40	95.2	2	4.8	0	0.0	79	91.9	1	1.2	6	7.0	0.08
Do interpersonal communication skills belong only to psychiatry?	3	7.1	39	92.9	0	0.0	0	0.0	85	98.8	1	1.2	0.03

At least 59.5% of registrars and 43.7% of specialists have been directly observed by senior colleagues whilst in training, during history taking, information giving and counselling. Both groups agreed that the feedback given was helpful.

Table 3 shows an overwhelming majority in both groups agreeing that communication training should be offered in neurology and be a core competency requirement in the neurology curriculum. However, there was not a strong support for testing of communication in the final FC Neurol board exit exam.

**TABLE 3:** Opinions relating to communication training during registrar training.

Variable	Yes		No		Unsure		Fisher’s exact score *p*
	*N*	%	*N*	%	*N*	%	
**Should communication training be offered in neurology?**							
Registrar	38	92.7	1	2.4	2	4.9	-
Specialist	76	91.6	2	2.4	5	6.0	-
Total	114	-	3	-	7	-	1.0
**Should communication be a core competency requirement?**							
Registrar	32	76.2	4	9.5	6	14.3	
Specialist	54	63.5	19	22.4	12	14.1	
Total	86	-	23	-	18	-	0.2
**Should communication be tested in FC Neurology exam?**							
Registrar	12	29.3	20	48.8	9	22	
Specialist	27	32.1	35	41.7	22	26.2	
Total	39	-	55	-	31	-	0.8

Of the 114 (88.4%) participants who felt that communication training should be offered during neurology training, registrars were more supportive of training in the first year of the registrarship whilst specialists felt that communication training should occur in all years of training. Role playing, videos and lectures were supported mostly by specialists, and on-the-job training was recommended by both groups more so than workshops and online study. In addition, other suggestions for the type of training included bedside teaching, direct observation by a consultant, interpersonal practical discussions, podcasts containing quizzes, practice with supervision, real-life mentoring, using recommended textbooks, sitting in with psychologists or psychiatrists during counselling, on-the-job training and small group discussions. These suggestions were mainly by specialists rather than registrars (14 vs. 1, respectively). Three other training formats were suggested and included podcasts with quizzes, biannual scheduled sessions of 90 min each and fortnightly or monthly tutorials or lectures.

Thirty-nine participants (30.2%) felt that communication should be formally tested in the neurology specialist exam mostly by means of an OSCE station in the clinical exam. In addition, there was good support for a workshop offering certification at the end of training. Three specialists recommended ongoing and continuous assessment during all years of registrar training.

**FIGURE 3:**
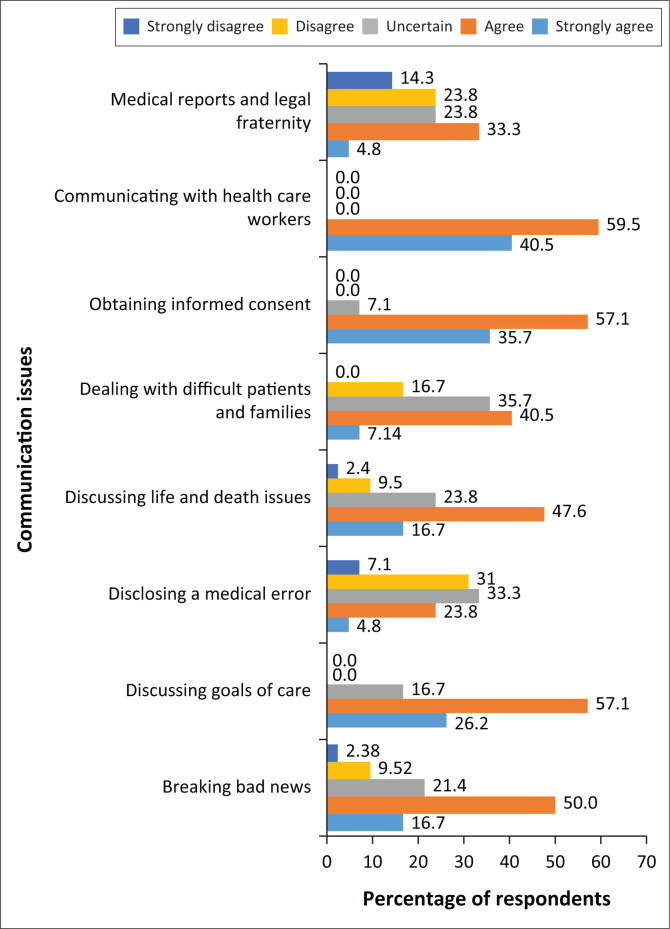
Registrars’ self-perceived competency in dealing with neurological communication issues in neurology.

**FIGURE 4:**
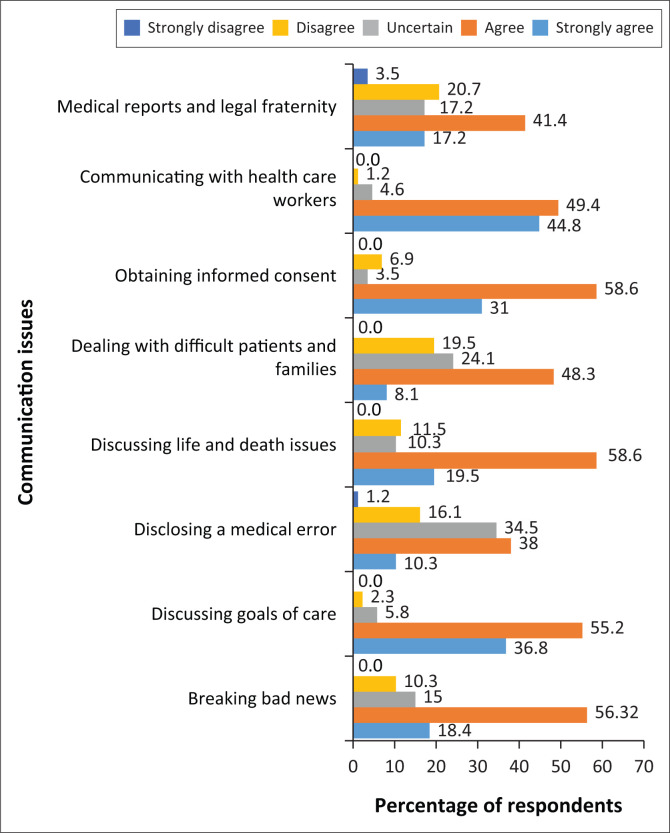
Neurology specialists’ self-perceived competency in dealing with communication issues in neurology.

The response from both groups as regards competency in dealing with various communication issues in neurology showed a moderate belief that the participants were well skilled. In fact, over 60% of registrars agreed or strongly agreed that they felt competent in breaking bad news, discussing goals of care, obtaining informed consent, discussing life and death issues and communicating with other health professionals (Figure 3). They felt less competent in disclosing medical errors to patients, dealing with difficult patients and families, writing medical reports and communicating with legal professionals. Over 70% of neurologists, on the contrary, agreed or strongly agreed that they were competent in dealing with communication issues in neurology for most situations except disclosing medical errors, dealing with difficult patients and families, writing of medical reports and dealing with the legal fraternity (Figure 4).

## Discussion

The field of medicine presents difficult communication issues for many doctors. Disciplines such as oncology, neurology, rheumatology and geriatrics manage patients who are afflicted by chronic, debilitating and terminal diseases and present with challenging demands on communication skills.^1,2^ In the case of neurology, examples include informing young patients with multiple sclerosis that 50% of them will be wheelchair-bound for 15 years, telling middle-aged patients that they have motor neuron disease with a remaining lifespan of 5 years^11,12^ and informing previously active individuals that they have Alzheimer’s disease or Parkinson’s disease which is progressive and unremitting. Having the skills to address these challenging situations is not innate to specialists but requires a separate set of skills besides those of eliciting tendon jerks and testing pupil reflexes. The current training of neurology registrars does not cater for these challenges. The College of Medicine has not made communication a core competency requirement for most specialities and neither does it make provision or recommendation for an assessment in communication skills.^8^

The goal of this study was to motivate for communication training of registrars and postgraduate assessment by surveying the opinions of registrars and specialists on their self-perceived communication skills. The neurology experience was studied and presented as representative of the smaller specialities where trainer–trainee contact is more than in the larger disciplines of general surgery, internal medicine and paediatrics. It was important to include registrars’ perspectives as they are currently in training and perhaps have had more exposure to undergraduate communication teaching which has improved in the recent past. In addition, registrars are more *au fait* with the current workload of clinical services during their training and better positioned to offer opinion on available time for communication training. There were more females in the registrar cohort than males and more males in the specialist cohort than females (*p* < 0.05). The distribution is reflective of the change in policy of registrar recruitment in South Africa where preference is given to females to address the traditional gender disparity of specialist training.^13^ The gender distribution of registrars was valid as the response rate was 93% and response bias was therefore unlikely.

A greater number of consultants (neurology specialists) use a translator for patients, who speak a language other than English or Afrikaans, rather than registrars (92% vs. 56%, *p* < 0.0001). Despite this significant setback in neurology specialists, they still do not regard their language skills as a barrier to communication. Many subtleties are lost in translation, and both verbal and non-verbal language forms the foundation of communication. Breaking bad news to a patient in a language they do not understand and by using a translator does have a negative impact on the doctor–patient relationship and the therapeutic relationship between the two.^14,15^ South African medical schools have realised this obstacle and have made the teaching of a geographically relevant indigenous language compulsory in the pre-clinical years of undergraduate medical training.^16,17^

Only 39.1% of specialists and 42.9% of registrars considered their training of interpersonal communication as adequate. The majority of registrars (71.4%) received this training as undergraduates and mostly in the fourth year of training, whereas a minority of specialists (26.4%) received their undergraduate training in interpersonal communication (71.4% vs. 26.4%, *p* < 0.0001). The 26.4% of specialists indicated training across all undergraduate years, but they still reported that this training was insufficient. Both groups strongly indicated no postgraduate training in interpersonal communication (83.3% for registrars and 83.9% for specialists, *p* = 1.000). This is a massive setback for the training of neurologists in South Africa considering that neurology presents unique categories of communication demands, requiring specific communication skills by the neurologist. For instance, knowing how to communicate with a dementing patient about advanced care planning and being cognisant of the various limitations that dementia presents during communication requires erudite communication skills. Knowing that patients with Alzheimer’s disease have poor working memory and are likely to forget recent events, although patients with semantic dementia have problems with language comprehension, presents different communication challenges even in patients collectively classified as demented.

Undergraduate training was in the form of role playing and lectures, but mostly concentrated on the doctor–patient communication for history taking and information giving (Figure 2). The challenging issues of communication requiring more introspection and speciality-specific issues were not covered during undergraduate communication training. This is understandable considering that undergraduate communication training is generic and not targeted at specialities.

Registrars were formally assessed in interpersonal communication at undergraduate level in the form of role playing and written exams. Specialists, on the contrary, were only tested in 30.2% of cases and this difference was statistically different. Similar testing procedures were used for both groups. In addition, registrars were tested at OSCEs. Given that assessment drives learning, it is clear from this statistic that the change in the undergraduate curriculum necessitating the training of communication is starting to show benefit. Table 2 shows that undergraduate training in communication did prepare registrars for practice in 53.3%, but in only 26.1% of specialists (*p* = 0.09), thus showing a trend but not statistical significance. Both groups feel competent in dealing with neurology communication issues in just over 50.0% of responders but are in strong agreement (registrars 95.2%, specialists 91.9%) that formal training in interpersonal communication is important. Specialists need communication skills and both groups were in agreement that such skills are not the prerogative of psychiatry alone.

During registrar training, acquisition of communication skills has largely been through experience, by observation of more senior colleagues and by being directly observed by senior colleagues when clerking and counselling patients. Unfortunately, this was only experienced by 59.5% and 43.7% of registrars and specialists, respectively. Notably, when feedback was given by senior colleagues, this was usually very helpful, thereby suggesting that on-the-job training is a useful strategy for communication skills learning, but this needs to be better formalised to be consistent and measureable.

The overwhelming majority are in agreement that communication training should be offered at postgraduate level and that it should be a core competency requirement. However, when it comes to testing of communication, both groups are reluctant to have this as an essential component in assessment with only 29.3% and 32.1% of registrars and specialists, respectively, being supportive. This is not unexpected as human nature would dictate that including an extra component for assessment is unlikely to be supported by the exam candidate. This responsibility falls on the shoulders of the college of medicine.

In terms of the need for communication training, specialists were in favour of training during all years of registrar training, in the form of role playing, videos, annual workshops, online self-study and mostly on-the-job training. Registrars, on the contrary, felt that most training should be conducted in the first year of registrar training, also via role playing and on-the-job training. It is conceivable that the added burden of the MMed dissertation which has become an HPCSA registration requirement for all registrars since 2011 has influenced this decision. Registrars feel pressured during their final years to complete their MMed dissertations and prepare for the fellowship exam. Communication training although essential is therefore preferred during the earlier years of training. This opinion is inclined to change if communication skills testing becomes a requirement to pass the fellowship exit exams.

Of those in support of communication being assessed in the specialist exams, 81.5% of neurologists and 50.0% of registrars felt that this should be tested at an objectively structured practical exam (OSPE). The Canadian neurology board exam allocates one of the 10 stations to communication where difficult and challenging communication issues in neurology are tested. This serves to fulfil many CanMEDS requirements for training. When the AfriMEDS framework is formally adopted by the college of medicine of South Africa (SA), the requirement for a communication component in the exit exams will more than likely be included.^5^ The AfriMEDS framework was adopted from the CanMEDS framework where the holistic doctor is expected to fulfil various roles in practice.^18^ These roles include being an expert health practitioner, communicator, collaborator, leader, health advocate, scholar and professional.

The self-perceived competency skills in various communication issues revealed unexpectedly high assessment values by registrars and specialists (Figures 3 and 4). The response is very subjective and not in keeping with the predominant theme for the need for more communication skills training as reported throughout the survey. Regardless of this, registrars and specialists agree that they have poor skills in disclosing a medical error, dealing with difficult patients and families and especially in writing medical reports and communicating with the legal fraternity. In fact both groups scored the latter very poorly, suggesting a critical area of need. According to Storstein and Jayalakshmi et al., learning how to break bad news and dealing with the legal professional on medical matters require special training presented by experts in the field.^19,20^

## Conclusion and recommendations

Given that assessment drives learning,^21^ it is possible that the need for communication training for neurologists can constructively be addressed if the college of medicine includes communication skill testing in the specialist exit exams. This will drive the need for training in communication during the specialist training period. The neurology experience shows us a dire need for communication skills teaching during postgraduate training and for specialists who are already in practice. A summative assessment of communication in the form of a testing station in an objectively structured clinical or practical exam should be considered for all specialities, if not included already. Departments that are currently equipped for communication training, such as the health professions education departments, family medicine, palliative medicine, psychology and psychiatry, should be recruited to support communication teaching for all other medical specialities.

## Figures and Tables

**FIGURE 1-A1:**
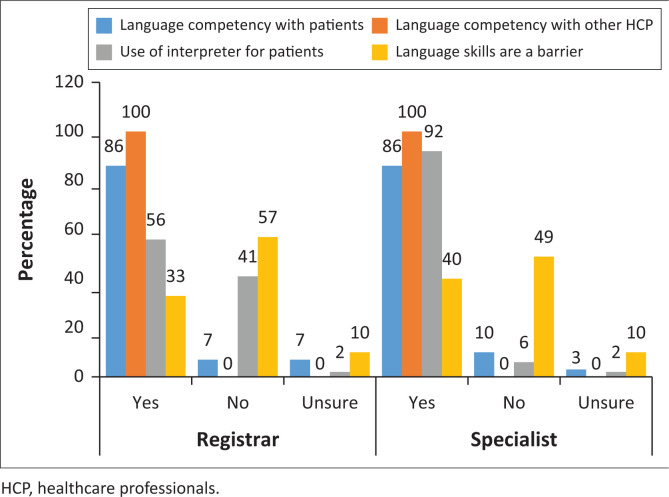
Language skills with patients and other healthcare professionals.
